# Fear of Childbirth in Nulliparous Women

**DOI:** 10.3389/fpsyg.2022.923819

**Published:** 2022-07-12

**Authors:** Yvette M. G. A. Hendrix, Melanie A. M. Baas, Joost W. Vanhommerig, Ad de Jongh, Maria G. Van Pampus

**Affiliations:** ^1^Department of Obstetrics and Gynecology, OLVG, Amsterdam, Netherlands; ^2^Department of Obstetrics and Gynecology, Martini Hospital, Groningen, Netherlands; ^3^Department of Research and Epidemiology, OLVG, Amsterdam, Netherlands; ^4^Academic Centre for Dentistry Amsterdam (ACTA), University of Amsterdam, VU University Amsterdam, Amsterdam, Netherlands

**Keywords:** fear of childbirth, pregnancy, delivery, help, pregnancy-related anxiety, gestation, nulliparous

## Abstract

**Purpose:**

The relation between fear of childbirth (FoC) and gestational age is inconclusive, and self-reported need for help regarding this fear has never been investigated. This study aimed to determine the prevalence and course of FoC according to gestational age, to identify risk factors for the development of FoC, the influence of this fear on preferred mode of delivery, and self-reported need for help.

**Methods:**

Nulliparous pregnant women of all gestational ages completed an online survey. The study consisted of a cross-sectional and a longitudinal analysis. Women who completed the survey in the first or second trimester (T_0_) were approached again in their third trimester (T_1_). The Wijma Delivery Expectancy Questionnaire Version A (W-DEQ A) was used with a cut-off score ≥ 85 to define presence of fear of childbirth. Questionnaires indexing social support, anxiety, symptoms of depression, preferred mode of delivery, and self-reported need for help were included.

**Results:**

In total, 364 women were enrolled at T_0_, and 118 out of 184 eligible women were included in the longitudinal analysis. Point prevalence of FoC at T_0_ was 18.4% with no significant difference between trimesters. In the longitudinal sample, the prevalence of FoC decreased from 18.6% (T_0_) to 11.0% (T_1_), *p* = 0.004. Although mean scores for FoC decreased significantly, *p* < 0.001, scores increased in 41 (34.7%) women. The presence of FoC was associated with elevated anxiety, less family support, prenatal care of the obstetrician by choice, preference for a cesarean section, and for pain relief. Women with FoC were more likely to actively seek for help compared to women without FoC.

**Conclusion:**

While FoC is common in each trimester, prevalence decreases over the course of pregnancy. Women with FoC are often actively seeking for help, suggesting that this fear should be addressed better, and help should be offered accordingly.

## Introduction

Prevalence rates of fear of childbirth (FoC) differ across countries and measurement methods, ranging from 4 to 20% (O’Connell et al., 2017; Nilsson et al., 2018). Literature on the etiology of FoC in nulliparous women suggests that its multifactorial cause of anxiety, depression, and low social support is found to be related to FoC (Storksen et al., 2012; Jokić-Begić et al., 2014; Lukasse et al., 2014; Dencker et al., 2019). Thus, FoC is a common fear among pregnant women with some women being more vulnerable to the development of FoC than others.

Fear of childbirth could influence preferred and actual mode of delivery as well as psychological well-being in the postpartum period. Pregnant women with FoC have been found to be more likely prefer epidural analgesia in a vaginal delivery and a planned cesarean section (CS; Haines et al., 2012; Raisanen et al., 2014a; Ryding et al., 2016; Sitras et al., 2017). Yet, CS rates differ across countries, including views on performing cesarean sections upon maternal request without medical necessity (Habiba et al., 2006; Betrán et al., 2016). This may explain why some studies found FoC to be related to a longer labor duration (Adams et al., 2012) and a greater likelihood of an (unplanned) CS (Laursen et al., 2009; Ryding et al., 2015; Takacs et al., 2019), whereas other studies could not detect a relation between FoC and (preferred) mode of delivery (Sluijs et al., 2012; Jespersen et al., 2014). Regarding the postpartum period, a meta-analysis found that FoC in pregnancy was associated with postpartum posttraumatic stress disorder (Ayers et al., 2016). Hence, since FoC could lead to adverse physical and psychological outcomes during delivery and the postpartum period, identifying and treating FoC in pregnancy is important.

To find the optimum time to identify and treat women with FoC, knowledge about the course of FoC over time is important. However, regarding the course of FoC during pregnancy, literature shows conflicting results (Huizink et al., 2004; Laursen et al., 2008; Hildingsson et al., 2011, 2017; Rothenberger et al., 2011; Richens et al., 2019). Longitudinal studies found either that FoC decreased (Huizink et al., 2004; Hildingsson et al., 2017) or increased (Hildingsson et al., 2011) as pregnancy progressed. Also, no relation between FoC and gestational age (Rothenberger et al., 2011) or conflicting patterns between women have been observed (Laursen et al., 2008; Richens et al., 2019). Since the course of FoC during pregnancy seems to differ across studies, the relation between FoC and gestational age is inconclusive.

Currently, there is no uniform guideline on screening for FoC in pregnancy. In addition, it is unclear whether pregnant women feel they are adequately provided with information about, and help with, FoC and whether women would prefer to receive additional help. To date, no studies have examined self-reported need for help in relation to FoC. If it proves to be the case that women themselves would like to receive additional help apart from the already provided pregnancy care, this would justify implementing help for FoC more standardly in the care than it is currently being done. Inherently, it is then also important to know when to screen for FoC and to provide this help. Therefore, the purpose of the present study was to determine the prevalence and course of FoC according to gestational age in nulliparous women and to evaluate self-reported need for help. We formulated the following main research questions. First, what is the prevalence and course of FoC in nulliparous pregnant women? And secondly, do nulliparous pregnant women express a need for help for FoC? Furthermore, since the literature has found multiple risk factors and consequences of FoC, we aimed to identify factors associated with FoC, and influence of FoC on the preferred mode of delivery.

## Materials and Methods

This observational survey study consisted of a cross-sectional analysis and subgroup longitudinal analysis among a convenience sample of pregnant women. Women were recruited from February 2019 to January 2020 through a city hospital (OLVG) and several midwifery practices in Amsterdam, Netherlands. Nulliparous women received a flyer about the study after their appointment with their obstetrician or at the midwifery practice.

In the Netherlands, obstetric care is divided between community midwifes (primary care), obstetrician-gynecologists (secondary care), and academic referral centers (tertiary care). To distinguish which care is needed, risk selection takes place based upon a national list of recommendations [List of Obstetric Indications; College voor zorgverzekeringen (College for Health Insurance), 2003]. For women with a low-risk profile, a community midwife can provide care. When needed, women are referred to an obstetrician. Women can also choose secondary care without medical necessity.

The flyer included information about the study, the inclusion criteria, and the address for the study website where they could receive more detailed information.^1^ Furthermore, pregnant women were recruited through social media such as pregnancy websites, LinkedIn, Facebook, and Instagram where information about the study and the URL to the study website was also provided.

The website of the study contained the patient information and the informed consent form. When interested in participating in the study, women entered their personal e-mail address on the study’s website. The personal e-mail address was then sent to one of the authors (YH) *via* a password-secured data file. Bias from possible repeated entry was prevented by ensuring e-mail addresses were not identical. The women were subsequently sent a personalized link to the online questionnaire (T_0_) through Castor EDC (Castor, 2019). The questionnaire could be completed on any electronic device that was connected to the internet and there was no specific time limit to complete the survey. There was no interference from the researcher during this time. Once fully completed, the answers could not be changed. There were no printed questionnaires used. Women who completed the questionnaire in their first or second trimester received an e-mail with a personalized link around the 35th gestational week to complete the same questionnaire(s) once more (T_1_). Eligible for the study were nulliparous women of all gestational ages. Exclusion criteria were multiparous women (defined as a previous pregnancy of ≥16 weeks), women who were younger than 18 years old, or who did not speak the Dutch language. Incomplete questionnaires were excluded.

The questionnaire included questions regarding demographics and obstetrical characteristics [i.e., age, gestational age, country of birth, partner, care led by midwife/gynecologist by choice/gynecologist for medical reasons, self-reported (history of) physical health, (history of) psychological treatment, medication use, previous pregnancies less than 16 weeks, fertility treatment, planned pregnancy, pregnancy complications, preferred mode and place of birth], validated questionnaires for FoC, anxiety, depression, social support as well as questions pertaining to need for help. The questions on socio-demographic background factors and obstetrical characteristics were pretested and validated by subject matter experts including gynecologists (in training), psychologists and a psychiatrist. Since the other questionnaires used were well validated, we did not pretest those questions.

Fear of childbirth was assessed using the W-DEQ Version A (Wijma et al., 1998). The W-DEQ A is a 33-item self-report questionnaire measuring FoC scored on a six-point Likert scale. Total scores vary from 0 to 165, with higher scores indicating higher FoC. The W-DEQ A has good psychometric properties with a high internal consistency (Cronbach’s alpha ≥0.87; Wijma et al., 1998). A cut-off score of ≥85 to indicate clinically relevant FoC has been mostly used and recommended (Wijma et al., 1998; Calderani et al., 2019). It has been translated to Dutch (Wijma and Wijma, 2004).

Symptoms of anxiety and depression were measured using the Hospital Anxiety and Depression Scale (HADS), a validated self-report questionnaire consisting of 14 items to measure anxiety and depressive symptoms (Zigmond and Snaith, 1983). Each item can be scored from 0 to 3 using a score range from 0 to 21 on each subscale with higher scores indicating more symptoms. It has been translated and validated in the Dutch population with a Cronbach’s alpha of 0.84 and 0.79 for the anxiety and depression scale, respectively (Spinhoven et al., 1997).

Social support was measured using the Multidimensional Scale of Perceived Social Support (MSPSS) which is a validated 12-item self-report questionnaire measuring support from family, friends, and a significant other (Zimet et al., 1988). Each item is scored from 1 to 7 with higher scores indicating more social support. The MSPSS has been validated in the pregnant population, with Cronbach’s alpha varying from 0.90 to 0.94 for the different subscales (Zimet et al., 1990). It has been translated and validated in the Dutch population (Pedersen et al., 2009).

Self-reported need for help was explored by one or multiple questions, depending on whether the woman was already receiving help. The question for need for help was designed using Prochaska’s stages of behavioral change (Prochaska et al., 1994), namely: ‘For fear of childbirth *(“I do not need extra guidance right now, I have no problems at the moment”* vs. “*I am still doubting if I want extra guidance”* vs. “*I’m actively searching for extra help”* vs. “*I am currently receiving extra help” vs “I have already received and completed help for fear of childbirth”)*. If the woman answered “*I do not need extra guidance right now, I have no problems at the moment*’ no follow-up question was asked. Otherwise, participants were asked about their preference for the type of health professional and timing of additional help.

### Statistical Analyses

Before analyzing the data, women were divided into three categories according to the gestational trimester (i.e., 0–12 weeks; 13–27 weeks; 28–42 weeks). Scores on the W-DEQ A were dichotomized using a cut-off score of ≥85 to determine the presence of FoC (Wijma et al., 1998). Descriptive statistics were used to describe socio-demographic and clinical data in absolute numbers and percentages. Groups were divided according to the presence of FoC yes/no. A Mann–Whitney U test was conducted comparing age and gestational age. A Chi-square test was used to compare categorical variables. The effect of gestational age on FoC was determined by performing a one-way ANOVA and Pearson’s Chi-square test. For the longitudinal analyses, change in W-DEQ A scores was analyzed by a paired samples t-test. A McNemar analysis was performed to analyze within-patient differences in prevalence rates over time. Logistic regression analyses were performed to identify potential predictors for the course of FoC (increase vs. decrease in scores on FoC). To assess potential significant variables and confounders, univariable regression analyses were carried out while potential risk factors for FoC, the preferred mode of delivery (vaginal birth or cesarean section, and pain relief), and preferred place of birth (at home or in the hospital) were determined using multivariable logistic regression analyses. To explore self-reported need for help for FoC, a separate logistic regression analysis was performed using the question on the need for help as a predictor of the presence of FoC. Only complete surveys were included in the analyses so there were no missing data.

Drop-out analyses were conducted to research potential differences between responders and drop-outs at T_0_ and T_1_ (i.e., lost to follow-up or incomplete questionnaires). Means and standard deviations are given as (M ± SD). When data were not normally distributed, median and interquartile ranges are presented. Statistical differences were indicated as *p* < 0.05 (reported *P* is two-sided). Results of regression analyses are presented as unadjusted and adjusted odds ratios (OR) with a 95% confidence interval. All analyses were performed with the Statistical Package for Social Sciences (SPSS) version 24.0 (Corp, 2016).

### Ethical Approval

Informed consent was obtained by actively checking a box agreeing to participate in the study, which was obligatory, and thereafter leaving their personal e-mail address. Furthermore, participants were provided an option to download the patient information and informed consent form. This study was exempted from ethical approval by the Medical Research Ethics Committees United (MEC-U) in Nieuwegein, Netherlands (reference number W18.188).

## Results

**Figure 1:**
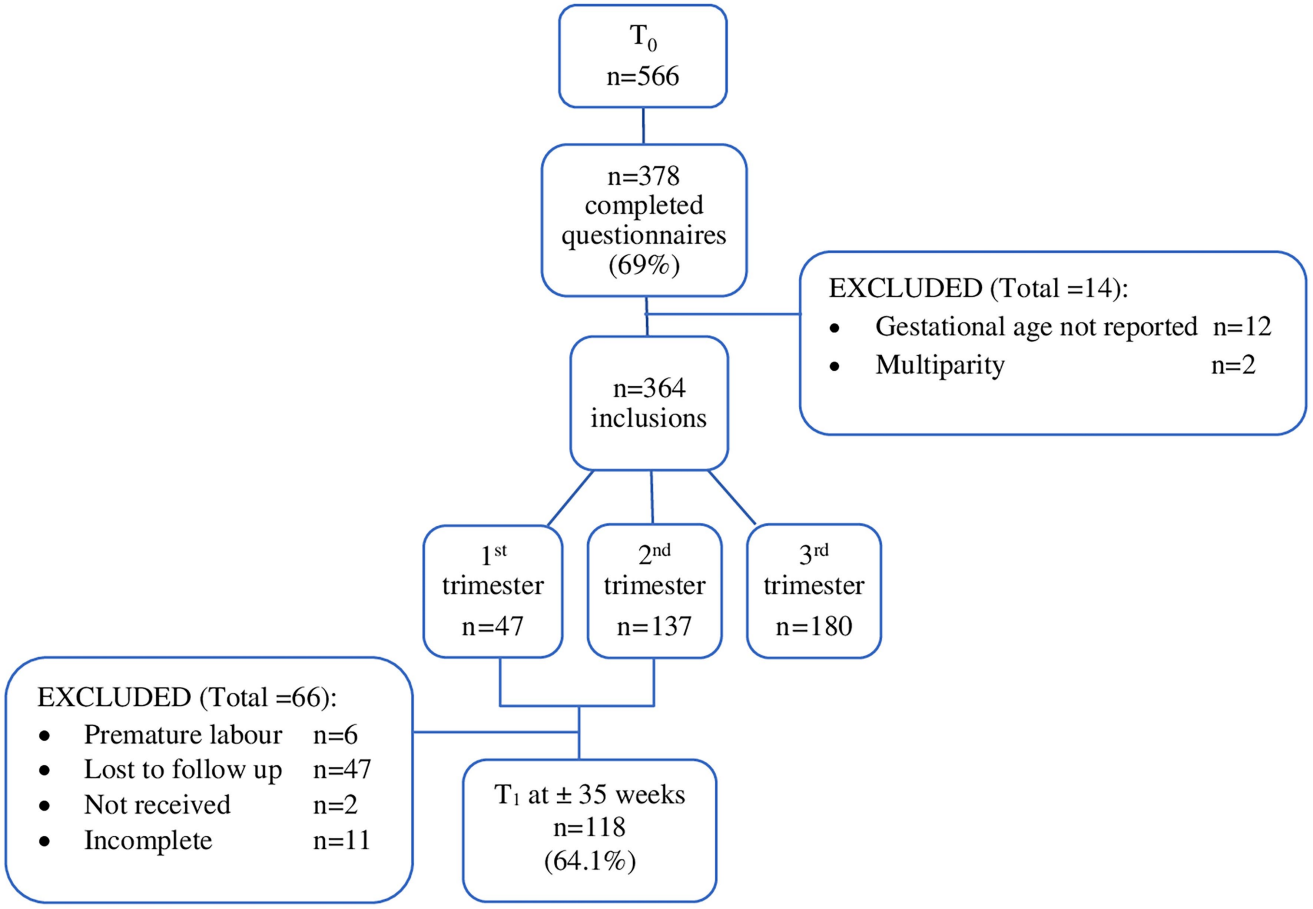
Flow diagram participants. Flowchart of participants. For the longitudinal sample, two participants (i.e., “not received”) did not receive a questionnaire at T_1_ because of a technical issue.

In total, 566 women agreed to participate, of which 378 (66.8%) completed the questionnaire at T_0_. Fourteen women were excluded because of unknown gestational age (*n* = 12) or being multiparous (*n* = 2), leaving 364 women to be included in the analyses. Of them, 184 (50.5%) received a follow-up questionnaire in their third trimester of pregnancy (T_1_) that was completed by 118 women (118/184, 64.1%; Figure 1). Background characteristics are shown in Table 1. Compared to women without FoC, women with FoC were more likely to receive care by a gynecologist by choice (*p* = 0.020), more often had a history of psychological treatment (*p* = 0.031), or were currently receiving psychological treatment (*p* = 0.009).

**Table 1:** Socio-demographic and obstetric characteristics compared per group.

Characteristics	Total *n* = 364	FoC *n* = 67 (18.4%)	No FoC *n* = 297 (81.6%)	Value of *p*
*Maternal age (years), median (range)*	30 (28–33)	31 (28–35)	30 (28–33)	0.082
*Gestational age (weeks), median (range)*	27 (17–35)	26 (16–33)	28 (17–35)	0.373
Trimester *n* (%)				0.406
First trimester	47 (12.9)	8 (11.9)	39 (13.1)	
Second trimester	137 (37.6)	30 (44.8)	107 (36.0)	
Third trimester	180 (49.5)	29 (43.3)	151 (50.8)	
*Educational level^a^ n (%)*				0.315
Low	10 (2.7)	1 (1.5)	9 (3,0)	
Middle	93 (25.5)	13 (19.4)	80 (26.9)	
High	261 (71.7)	53 (79.1)	208 (70.0)	
*Country of birth n (%)*				0.069
The Netherlands	343 (94.2)	60 (89.6)	283 (95.3)	
Other	21 (5.8)	7 (10.4)	14 (4.7)	
*Partner status n (%)*				0.487
No partner	10 (2.7)	1 (1.5)	9 (3.0)	
Partner	364 (97.3)	4 (98.5)	288 (97)	
*Care led by n (%)*				**0.020**
Midwifery practice	264 (72.5)	43 (64.2)	221 (74.4)	
Gynecologist (choice)	16 (4.4)	7 (10.4)	9 (3.0)	
Gynecologist (medical)	84 (23.1)	17 (25.4)	67 (22.6)	
*Self-reported health n (%)*				0.820
Healthy	340 (93.4)	63 (94.0)	277 (93.3)	
Not healthy	24 (6.6)	4 (6.0)	20 (6.7)	
*History of physical illness n (%)*				0.978
No	245 (67.3)	45 (67.2)	200 (67.3)	
Yes	119 (32.7)	22 (32.8)	97 (32.7)	
*Medication n (%)*				0.349
No	287 (78.8)	50 (74.6)	237 (79.8)	
Yes	77 (21.2)	17 (25.4)	60 (20.2)	
*History of psychological treatment n (%)*				**0.031**
No	190 (52.2)	27 (40.3)	163 (54.9)	
Yes	174 (47.8)	40 (59.7)	134 (45.1)	
*Current psychological treatment n (%)*				**0.009**
No	337 (92.6)	57 (85.1)	280 (94.3)	
Yes	27 (7.4)	10 (14.9)	17 (5.7)	
*History of Previous pregnancy under 16 weeks n (%)*				0.133
No	249 (68.4)	51 (76.1)	198 (66.7)	
Yes	115 (31.6)	16 (23.9)	99 (33.3)	
*Fertility treatment n (%)*				0.544
No	312 (85.7)	59 (88.1)	253 (85.2)	
Yes	52 (14.3)	8 (11.9)	44 (14.8)	
*Planned pregnancy n (%)*				0.238
No	49 (13.5)	12 (17.9)	37 (12.5)	
Yes	315 (86.5)	55 (82.1)	260 (87.5)	

To compare groups (FoC yes/no) Chi-square tests and Mann–Whitney U tests were performed for categorical and continuous variables, respectively. ^a^Educational levels: low = primary school and preparatory secondary education; middle = senior general secondary education, secondary vocational education and pre-university education; high = higher professional education and university education. Bold values are statistically significant (p < 0.05).

Analyses on differences in demographics between completers and non-completers at T_0_ showed that women who completed the questionnaire more often reported a history of psychological treatment (47.8 vs. 30.2%), *p* = 0.029, and a high level of education (71.7 vs. 40.4%) than the non-completers, *p* < 0.001. No other demographic variables were statistically significantly different between the completers and non-completers.

### Fear of Childbirth

The overall prevalence rate of FoC was 18.4% (*n* = 67) and did not differ across trimesters *χ*^2^ (2, *N* = 364) = 1.80, *p =* 0.406. In the total cross-sectional sample, the mean sum score on the W-DEQ A (65.8 ± 23.0) did not significantly differ across trimesters, *F* (2, 361) = 0.36, *p* = 0.700. For women with FoC in the cross-sectional sample, a significant difference in mean scores across trimesters was found (*F*, 2, 64) = 3.67, *p* = 0.031); a Tukey post-hoc test revealed that mean score was significantly lower for women in their third trimester compared to women in their first trimester (Table 2).

**Table 2:** Means, standard deviations and One-Way ANOVA on scores on the W-DEQ A.

	W-DEQ A scores per trimester												
Group	First		Second		Third		Total		ANOVA			Tukey’s HSD comparisons^a^	
	*n* (%)	M ± SD	*n* (%)	M ± SD	*n* (%)	M ± SD	*n* (%)	M ± SD	df	*F*-ratio	Value of *p*		Value of *p*
Total	47 (12.9)	66.9 ± 28.9	137 (37.6)	66.8 ± 24.0	180 (49.5)	64.8 ± 20.5	364 (100)	(65.8 ± 23.0)	2	0.36	0.700	First vs. second	0.080
FoC	8 (11.9)	112.6 ± 20.3	30 (44.8)	99.8 ± 15.7	29 (43.3)	96.7 ± 11.8	67 (18.4)	(100.0 ± 15.3)	2	3.67	**0.031**	First vs. third	**0.023**
No FoC	39 (13.1)	57.6 ± 20.1	107 (36.0)	57.6 ± 16.7	151 (50.8)	58.7 ± 15.6	297 (81.6)	(58.1 ± 16.6)	2	0.16	0.849	Second vs. third	0.704

ANOVA = analysis of variance, df = degrees of freedom. ^a^Post-hoc analysis for women with FoC since overall ANOVA showed a statistically significant difference in score on the W-DEQ A across trimesters.Bold values are statistically significant (p < 0.05).

### Longitudinal Group

In total, 118 out of 184 eligible women (64.1%) completed the questionnaire again around the 35th week of pregnancy (T_1_). Two women did not receive the questionnaire due to a technical issue. Women who completed the questionnaire at T_1_ significantly more often reported a high educational level (*n* = 97, 82.2%) than drop-outs (*n* = 33, 56.9%), *χ*^2^ = 13.746, *p* = 0.001. The W-DEQ A mean score at T_0_ was not significantly different between completers (65.6 ± 26.6) and drop-outs (68.4 ± 23.6), *t*(174) = 0.691, *p* = 0.347. No significant difference in the prevalence of FoC at T_0_ between completers (*n* = 22, 18.6%) and drop-outs (*n* = 13, 22.4%) was found, *χ*^2^ = 0.347, *p* = 0.556.

The proportion of women with FoC decreased from 18.6% (22/118) at T_0_ to 11.0% (13/118) at T_1_, *p* = 0.004. For the total group, the group with FoC at T_0_ and without FoC at T_0_, W-DEQ A mean scores significantly decreased over time (Table 3). Individually, an increase in score on FoC was found in 41 (37.4%) women, with a mean increase of 8.1 ± 7.1 points on the W-DEQ A. Of those women, 37 women had a score below the cut-off of ≤85 at both time-points and four women had FoC at both time points. There were no women who developed FoC over time; all women who had FoC at T_1_, also had FoC at T_0_. No significant predictors for an increase in score on FoC over time were found.

**Table 3:** Changes in W-DEQ A scores in the longitudinal sample.

Group	n (%)	W-DEQ A over time (M ± SD)		Paired *t*-test		
		*T*_0_	*T*_1_	*t*-value	df	Value of *p*
Total group	118 (100)	65.6 ± 26.6	59.1 ± 24.2	4.356	117	<0.001
FoC at *T*_0_	22 (18.6)	106.3 ± 16.9	87.8 ± 27.7	3.774	21	0.001
No FoC at *T*_0_	96 (81.4)	56.3 ± 18.3	52.5 ± 17.7	2.855	95	0.005

Paired sample *t*-tests were done to compare scores on the W-DEQ A over time. df = degrees of freedom.

### Risk Factors and Mode of Delivery

Multivariable logistic regression analysis showed that a higher score on anxiety, lower social support from family, and choosing to be in medical prenatal care was associated with the presence of FoC (Table 4). FoC was significantly associated with a preference for a planned CS and for pain relief during delivery, but not with a preference for place of birth (Table 5).

**Table 4:** Multivariate logistic regression analysis on risk factors for FoC.

	FoC (+)	FoC (−)				
Variable	*n* = 67 *Mdn* (IQR) n (%)	*n* = 297 *Mdn* (IQR) n (%)	OR (95% CI)	Value of *p*	aOR (95% CI)	Value of *p*
Depression^a^	4 (2–9)	3 (2–5)	1.1 (1.0–1.2)	0.002	0.9 (0.8–1.0)	0.080
Anxiety^a^	8 (5–11)	4 (2–6)	1.3 (1.2–1.4)	<0.001	1.3 (1.2–1.5)	**<0.001**
Social support family^b^	5.5 (4.5–6.8)	6.5 (5.8–7.0)	0.6 (0.5–0.7)	<0.001	0.7 (0.6–0.9)	**0.003**
Social support friends^b^	6.0 (5–6.8)	6.5 (5.8–7.0)	0.7 (0.6–0.8)	<0.001	–	–
Social support SO^b^	6.8 (6.0–7.0)	7 (6.5–7.0)	0.7 (0.5–0.95)	0.023	–	–
*Care led by* ^c^						
Gynecologist (choice)	7 (10.4)	9 (3.0)	4.0 (1.4–11.3)	0.009	4.1 (1.2–13.5)	**0.020**
Gynecologist (medical)	17 (25.4)	67 (22.6)	1.3 (0.7–2.4)	0.405	–	–
Current psychological treatment^d^	10 (14.9)	17 (5.7)	2.9 (1.3–6.6)	0.012	1.3 (0.5–3.3)	0.950
History of psychological treatment^d^	40 (59.7)	134 (45.1)	1.8 (1.1–3.1)	0.032	0.9 (0.5–1.7)	0.800

Multivariate logistic regression analyses was performed to identify potential risk factors for FoC. OR = Odds ratio, aOR = adjusted odds ratio, CI = confidence interval. ^a^HADS. ^b^MSPSS, SO = significant other. ^c^Ref category = midwifery practice. ^d^Ref category = no.Bold values are statistically significant (p < 0.05).

**Table 5:** Preference for mode of delivery and self-reported need for help for FoC.

	FoC (+)	FoC (−)				
Variable	*n* = 67 n (%)	*n* = 297 n (%)	OR (95% CI)	*P*	aOR (95% CI)^a^	*P*
Preference for^a^						
Cesarean section^b^	16 (23.9)	9 (3)	10.0 (4.2–23.9)	<0.001	9.2 (3.5–24.4)	**<0.001**
*Pain relief* ^c^						
I do not know yet	28 (41.8)	133 (44.8)	2.7 (1.3–5.9)	0.01	3.1 (1.3–7.0)	**0.008**
Yes	29 (43.3)	34 (11.4)	11.1 (4.9–25.0)	<0.001	9.3 (3.7–23.5)	**<0.001**
Hospital birth^d^	57 (85.1)	202 (68.0)	2.7 (1.3–5.5)	0.007	2.1 (1.0–4.5)	0.06
*For fear of childbirth I (am)..* ^e^						
Do not need extra help	21 (31.3)	242 (81.5)	–	–	–	–
Doubting if I want extra help	21 (31.3)	36 (12.1)	6.7 (3.3–13.5)	<0.001	6.3 (3.0–13.0)	**<0.001**
Actively searching for help	13 (19.4)	8 (2.7)	18.7 (7.0–50.3)	<0.001	17.5 (6.4–47.7)	**<0.001**
Currently receiving help	12 (17.9)	6 (2.0)	23.0 (7.9–67.6)	<0.001	21.6 (7.3–64.4)	**<0.001**
Had extra help and completed guidance	0 (0)	5 (1.7)	0.000	0.999	–	–

Separate logistic regression analyses were performed for preferred mode of delivery and for self-reported need for help for FoC. OR = odds ratio, aOR = adjusted odds ratio, CI = confidence interval. ^a^aOR is adjusted for health care provider, current psychological problems or treatment, score on the HADS-Anxiety, and social support from family. ^b^Ref category = vaginal birth. ^c^Ref category = no pain relief. ^d^Ref category = home birth. ^e^aOR is adjusted for baseline characteristics history of psychological problems requiring treatment or current psychological treatment.Bold values are statistically significant (p < 0.05).

### Self-Reported Need for Help

Women with FoC were more likely to be still in doubt whether they wanted extra help, actively seeking for help, or already receiving extra help compared to women without FoC (Table 5). There was no statistically significant difference between the need for help and receiving care from a midwife, an obstetrician by medical necessity, or by choice, *χ*^2^ (4, *N* = 364) = 7.32, *p* = 0.120.

Help for FoC was most often wanted or received from a midwife (63.4%, *n* = 64), gynecologist (26.7%, *n* = 27), or psychologist (19.8%, *n* = 20). Preference for the timing of help for FoC was indicated as: “*As soon as possible”* in 15.4% (*n* = 12), “*Far before delivery (around 30–35 weeks gestational age)*” in 55.1% (*n* = 43), or “*Just before the delivery (after 35 weeks gestational age”* in 25.6% (*n* = 20). Three women (3.8%) answered “*Different timing*” but did not specify what the preferred timing of help was.

## Discussion

This study used both a cross-sectional as well as a longitudinal group, to research the prevalence and course of FoC during pregnancy. In support of our main hypothesis, results showed that FoC was common among women with an average prevalence rate of 18.4%. In our longitudinal sample, both prevalence and mean score of FoC significantly decreased over time. Further, the presence of FoC was found to be related to less family support, elevated anxiety, and prenatal care of the obstetrician by choice. Regarding the influence of FoC on preferred mode of delivery, women with FoC were more likely to prefer a cesarean section and pain relief, compared to those without FoC. Another important finding was that women with FoC were more likely to be actively seeking for help compared to women without FoC.

Our results on the prevalence and course of FoC are partially in line with previous literature (Huizink et al., 2004; Nieminen et al., 2009; Lukasse et al., 2014; Hildingsson et al., 2017; O’Connell et al., 2017; Fairbrother et al., 2018). The prevalence rate of FoC (18.4%) in the present study is somewhat higher than in a recent meta-analysis (O’Connell et al., 2017) albeit there is a wide variety in prevalence rates across countries. In line with previous studies (Nieminen et al., 2009; Lukasse et al., 2014; Fairbrother et al., 2018), we did not observe a relation between gestational age and FoC in the cross-sectional group overall. However, for women with FoC, we did see that women in their third trimester scored significantly lower on FoC than those in their first trimester. In the longitudinal sample, we found an overall decrease in prevalence and mean scores of FoC over the course of pregnancy. Yet, patterns differed individually with a minority of women showing an increase in scores over time while no predictors for this increase were found. Importantly, even though in some women scores on FoC increased, no women in our sample developed clinically relevant FoC over the course of their pregnancy, which is opposed to women in a previous study who did develop FoC over time as measured by the Fear of Birth Scale (Hildingsson et al., 2017). Furthermore, women either had clinically relevant FoC throughout their pregnancy, or FoC decreased to below the cut-off in the third trimester. This suggests that screening negative at the beginning of pregnancy may reduce the likelihood of developing FoC over time while the other way around is more plausible in that FoC decreases later on in pregnancy. Therefore, we suggest that women should be screened for FoC at the beginning of pregnancy. Next, women should be counseled about treatment options for FoC and the possibility of spontaneous recovery. If a woman chooses to wait with treatment, it is important to monitor FoC throughout pregnancy.

We found three risk factors to be related to FoC. Firstly, compared to women without FoC, women with FoC reported a higher level of general symptoms of anxiety, which is consistent with previous literature (Dencker et al., 2019). Secondly, less social support from family members was related to FoC, but this was not demonstrated regarding support from friends or significant others. A possible explanation for this finding might be the high level of overall social support in our sample; for instance, almost all participating women had a partner. Thirdly, women with FoC and an uncomplicated pregnancy more often chose to have prenatal care by an obstetrician. It could be that choosing prenatal care from an obstetrician might not be a risk factor for FoC, but rather reflecting a sense of security the hospital embodies, and thereby a consequence of FoC rather than a risk factor. In contrast to other studies (Laursen et al., 2008; Storksen et al., 2012; Dencker et al., 2019), we did not find a significant relation between FoC and symptoms of depression, which may be explained by the overall low scores on depression in our study.

Regarding the preferred mode of delivery, women with FoC were more inclined to have a preference for a cesarean section and for pain relief during delivery, which is also in line with previous literature (Nieminen et al., 2009; Sluijs et al., 2020).

Regarding our aim to evaluate self-reported need for help for FoC, we found that women with FoC were often actively seeking additional help. This suggests that discussing FoC with a healthcare professional may not be standard practice nor sufficient to help and support women with these problems. This notion is supported by a recent study that concluded midwives should acquire more in-depth knowledge about FoC (de Vries et al., 2020). Accordingly, it is important that after screening positive for FoC, women are referred to a trained specialist on FoC, preferably a psychologist.

Both strengths and limitations should be recognized. One strength is that this study is the first observational study with both a cross-sectional and a longitudinal sample to study the relation between FoC and gestational age in nulliparous pregnant women while using the validated W-DEQ A questionnaire to measure FoC. Secondly, asking women whether they would like to receive help for FoC provides in-depth insight into the perspective of pregnant women and willingness for treatment of FoC. Besides mentioning these strengths, some limitations need to be noted. No significant differences were found between trimesters on the W-DEQ A score of the total sample, albeit a post-hoc power analysis showed that with the current sample size and reported means, power was <15% to detect significant differences in this group. Yet, the statistical power to detect significant differences was 99% for our longitudinal sample. Secondly, while all other demographic variables were similar, drop-out analyses at T_0_ and T_1_ revealed that completers significantly more often had a history of psychological treatment (T_0_) and a high educational level (T_0_ and T_1_) in comparison to drop-outs, which may have led to attrition bias. Although efforts were made to create a diverse sample by recruiting women from multiple settings, compared to national data from the Netherlands, our sample was more often born in the Netherlands (94.2 vs. 76.8%; Central Bureau for Statistics, 2020a) and more often had a high educational level (71.7% vs. 53.6%; Central Bureau for Statistics, 2020b). These differences in country of birth and educational level may have led to an under-reporting of FoC. Namely, studies have found that risk factors for FoC include a low educational level (Laursen et al., 2008; Salomonsson et al., 2013; Ryding et al., 2015; Fairbrother et al., 2018) and being foreign born (Ternström et al., 2015). Therefore, prevalence may even be higher in the general pregnant population, underlying the importance of being attentive to FoC in pregnancy. However, other studies have not found such an association with educational level (Nieminen et al., 2009) or report a higher risk of FoC in women with a high educational level (Raisanen et al., 2014b). Future studies should aim to include a more diverse sample of pregnant women from remote areas and areas of low socioeconomic status, and to distribute a survey in multiple languages.

## Conclusion

Fear of childbirth appeared prevalent in almost one in five women in each trimester and may decrease over time while women expressed a need for help. This highlights the need for standardized care of FoC and research into the application of screening tools and evidence-based treatments for those suffering from FoC. When pregnant women present themselves to the obstetrician, a thorough evaluation of patients’ social system is recommended, and reasons for choosing medical care should be asked for while being attentive to women who suffer from general anxiety. Attention should be given to requests for delivery by a planned CS without the medical necessity to rule out the possibility of an underlying FoC. Given the combination of a high prevalence and self-reported need for help, our recommendation would be that women are routinely screened for FoC at the beginning of pregnancy. More obstetricians and midwives should be aware of what possible treatment options are and where to find these so that women can be guided.

## Data Availability Statement

The raw data supporting the conclusions of this article will be made available by the authors, without undue reservation.

## Ethics Statement

The studies involving human participants were reviewed and approved by Medical Research Ethics Committees United (MEC-U) in Nieuwegein, Netherlands. The patients/participants provided their written informed consent to participate in this study.

## Author Contributions

YH: conceptualization, methodology, formal analysis, investigation, resources, and writing—original draft. MB: conceptualization, resources, and writing—review and editing. JV: methodology and formal analysis. AJ: writing—review and editing and supervision. MP: conceptualization, writing—review and editing, and supervision. All authors contributed to the article and approved the submitted version.

## Conflict of Interest

The authors declare that the research was conducted in the absence of any commercial or financial relationships that could be construed as a potential conflict of interest.

## Publisher’s Note

All claims expressed in this article are solely those of the authors and do not necessarily represent those of their affiliated organizations, or those of the publisher, the editors and the reviewers. Any product that may be evaluated in this article, or claim that may be made by its manufacturer, is not guaranteed or endorsed by the publisher.

## Footnotes

^1^ www.olvg.nl/hear
